# Associative Learning Through Acquired Salience

**DOI:** 10.3389/fnbeh.2015.00353

**Published:** 2016-01-11

**Authors:** Mario Treviño

**Affiliations:** Laboratorio de Plasticidad Cortical y Aprendizaje Perceptual, Instituto de Neurociencias, Universidad de GuadalajaraGuadalajara, Mexico

**Keywords:** effective salience, acquired salience, acquired predictiveness, visual discrimination, associative learning

## Abstract

Most associative learning studies describe the salience of stimuli as a fixed learning-rate parameter. Presumptive saliency signals, however, have also been linked to motivational and attentional processes. An interesting possibility, therefore, is that discriminative stimuli could also *acquire* salience as they become powerful predictors of outcomes. To explore this idea, we first characterized and extracted the learning curves from mice trained with discriminative images offering varying degrees of structural similarity. Next, we fitted a linear model of associative learning coupled to a series of mathematical representations for stimulus salience. We found that the best prediction, from the set of tested models, was one in which the visual salience depended on stimulus similarity and a non-linear function of the associative strength. Therefore, these analytic results support the idea that the *net* salience of a stimulus depends both on the items' *effective* salience and the motivational state of the subject that learns about it. Moreover, this dual salience model can explain why learning about a stimulus not only depends on the *effective* salience during acquisition but also on the specific learning trajectory that was used to reach this state. Our mathematical description could be instrumental for understanding aberrant salience acquisition under stressful situations and in neuropsychiatric disorders like schizophrenia, obsessive-compulsive disorder, and addiction.

## Introduction

In nature, visual stimuli are organized in complex combinations. Animals must focus their visual system on salient objects from visual scenes to extract relevant information for guiding their behavior. The physical properties of the stimuli (*v.gr.* their structure or intensity) contribute to establishing how salient or conspicuous they are (Itti and Koch, 2001; Pearce and Bouton, 2001). Such *effective* salience specifies the relative capacity of a stimulus to stand out among other items in the visual scene. Consequently, salient stimuli attract attention and increase the rate of learning about them as well as other similar visual objects (Rescorla and Wagner, 1972; Mackintosh, 1975; Le Pelley, 2004; Treviño et al., 2013). From both behavioral and neurobiological perspectives, salience is a fundamental stimulus-specific learning rate parameter.

Associative learning theories propose that the increments of associative strength decrease linearly as a function of that value (Bush and Mosteller, 1951). Many models propose that the rate of conditioning also depends on stimulus salience, but they generally represent it as a fixed quantity that depends on the physical attributes of the stimulus (Rescorla and Wagner, 1972). Thus, from this point of view, the contribution of discriminative stimuli to learning would be fixed and determined, exclusively, by their *effective* salience. There is a body of literature, however, that reveals that this notion is incomplete. For example, learning about an item depends on the amount of visual attention that is paid to it (McFarland, 1971; Mackintosh, 1975; Ahissar and Hochstein, 1993; Jiang and Chun, 2001; Baker et al., 2004; Harris, 2006; Griffiths and Mitchell, 2008; Gutnisky, 2009; Roelfsema et al., 2010) and, also, pre-exposure to stimuli uncorrelated with reinforcement reduces their associability (Lubow and Moore, 1959). Thus, an interesting possibility would be that discrimination learning also involves dynamic changes in stimulus salience. Salience changes could be related to processes such as motivation, attention, and arousal (Mackintosh, 1975; Koch and Ullman, 1985; Desimone and Duncan, 1995; Kustov and Robinson, 1996; Reynolds et al., 2000). Indeed, some theories of selective attention propose that the salience of a stimulus is not only a fixed consequence of its *effective* salience, but it also varies with the subject's experience with it and other stimuli (*i.e., acquired* salience). Esber and Haselgrove (2011) proposed that the *net* salience of a stimulus can be represented by the sum of *effective* plus *acquired* components. From this perspective, the total salience would initially depend on the physical properties of the stimulus, but it would then also vary with experience affecting the way subjects direct their attention toward sensory stimuli (Mackintosh, 1975; Pearce and Hall, 1980; Le Pelley, 2004; Esber and Haselgrove, 2011). There are two main possibilities about how stimulus salience could evolve with learning. One option is that salience would increase if the stimulus “predicts reinforcement more accurately than other stimuli present in the situation” but it would decrease “if it predicts reinforcement less accurately” (Mackintosh, 1975). Alternatively, training stimuli could lose salience as they become better predictors of a consequence (Pearce and Hall, 1980).

We have explored and characterized recently some of the visual learning capacities of adult mice. We trained freely moving animals to learn to discriminate between one conditioned (CS^+^) and multiple non-conditioned (${\text{CS}}_{i}^{-}$) visual stimuli (*i.e.*, images), which offered different degrees of structural similarity (SSIM) with respect to the CS^+^ Wang et al., 2004; Treviño et al., 2013. We've found that the sign and slope of the SSIM gradients led to markedly different learning curves. More precisely, training with negative similarity gradients led to a faster learning rate and a higher (but less precise) average choice performance than with positive gradients (Treviño et al., 2013). Yet, although we made a detailed characterization of both discrete and continuous behavioral measures during learning (Treviño et al., 2013), we didn't explore whether and how the empirical learning curves could be described from the perspective of associative learning theories. Motivated by this question, we here tested a series of mathematical models aiming to predict the empirical learning curves from the mice trained with varying stimulus similarity (Treviño et al., 2013). We adapted a basic differential equation for associative learning coupled to an operator that defined stimulus salience in various relevant ways. Notably, from the set of tested models, we found that the best predictor was one in which we represented the *net* salience as the sum of *effective* plus *acquired* components, as previously suggested (Esber and Haselgrove, 2011). Thus, our analytic results strongly support the idea that the *net* stimulus salience can indeed vary as a function of the associative history of discriminative stimuli (Mackintosh, 1975; Pearce and Hall, 1980; Esber and Haselgrove, 2011; Treviño et al., 2013). One implication of this interpretation is that learning about discriminative stimuli depends on the specific *effective* salience trajectory used to learn about them. We thus propose that, at any given time, the external (*effective*) and internal (*acquired*) salience elements determine which predictive value will be assigned to conditioned stimuli (Treviño et al., 2011). Understanding how exactly the brain processes salience is a fundamental step to advance current associative learning theories. Verifiable predictive models could become crucial to understanding aberrant salience acquisition in stressful situations and pathological states like schizophrenia, obsessive-compulsive disorder, and addiction.

## Materials and methods

### Animals

**Figure 1:**
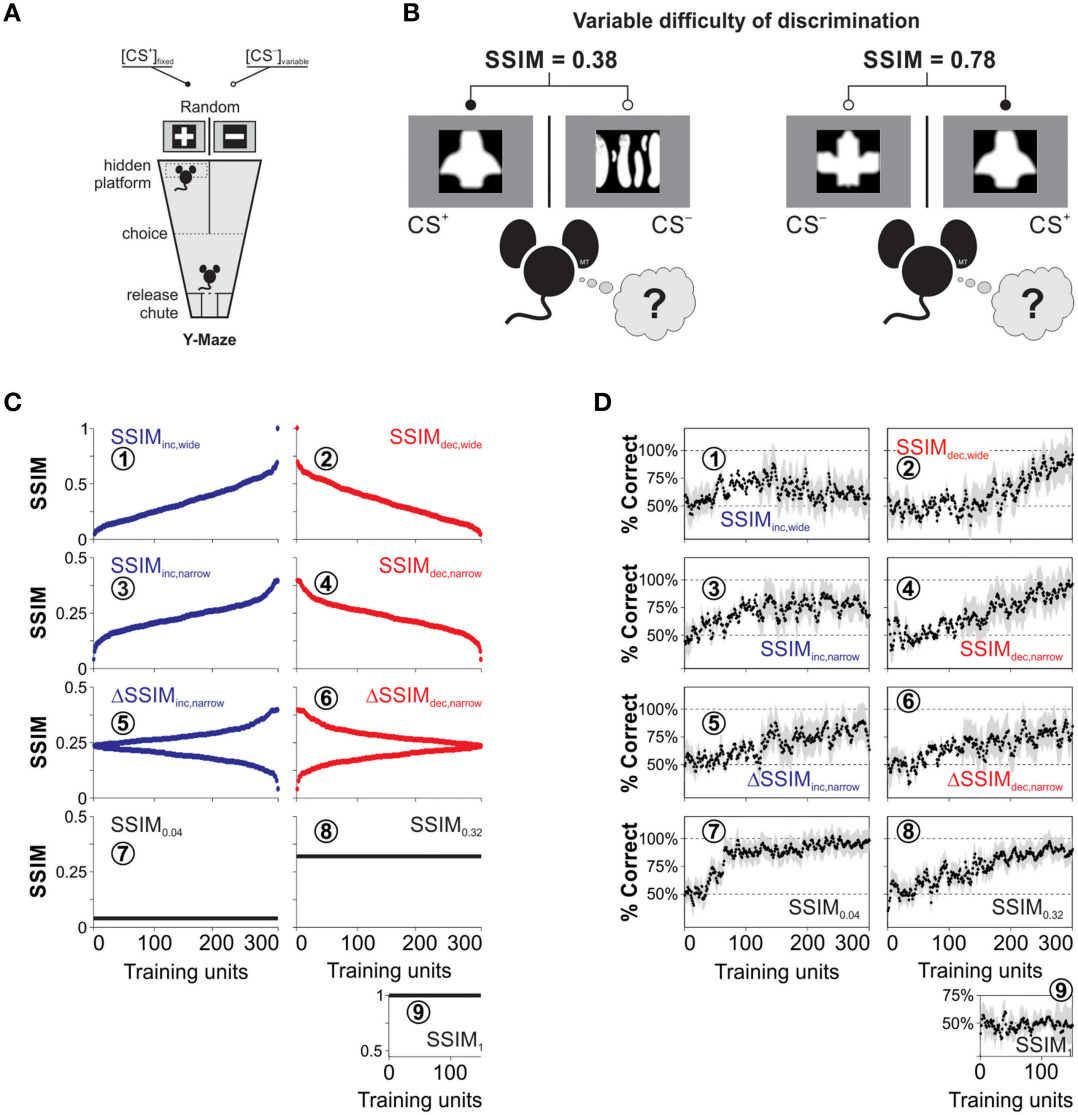
**Training paradigms and visual discrimination learning with heterogeneous stimulus similarity**. **(A)** Drawing of the visual discrimination task where two monitors are facing the ends of the arms of a Y-watermaze. They simultaneously display the conditioned (CS^+^) and non-reinforced (CS^−^) equiluminant stimuli (100% contrast). A submerged transparent platform below the CS^+^ serves as the unconditioned stimulus (US). The position of both the CS^+^ and the platform varies randomly on every trial. We placed the mice inside a release chute, and they learned to swim toward the CS^+^ because of the transparent platform positioned below it. **(B)** Sample CS^+^ stimulus with different CS^−^ stimuli during training trials. The difficulty of the discrimination task depends on the degree of structural similarity (SSIM) between images, indicated on the top. **(C)** CS^−^ stimuli can be arranged by increasing (blue dots), decreasing (red dots), or constant (black lines) similarity with respect to the CS^+^. **(D)** Corresponding empirical learning curves for the nine groups of mice trained with the corresponding SSIM programs.

We trained behaviorally naïve wild-type male mice (C57BL/6, *n* = 88, P40-50, Charles River) in a dichotomic visual discrimination task (Figure 1A). Mice were housed individually under a 12/12 h light/dark cycle with free access to food and water. Groups of 4–6 mice were trained during the light phase, 5 days a week. All animal experiments were carried out following the animal welfare guidelines of the Max Planck Society (G-171/10) and the Universidad de Guadalajara (SAGARPA, NOM-062-ZOO-1999), in accordance with the NIH's Guide for the Care and Use of Laboratory Animals. The experimental protocol was approved by the ethics comitee of the Instituto de Neurociencias, Universidad de Guadalajara, Mexico.

### Visual discrimination training

We trained the mice using a forced choice, swimming discrimination task in which the mice controlled their decision time autonomously (Treviño et al., 2013). We illustrate a schematic view of the visual swimming task in Figure 1A. In this task, the animals learned to associate that swimming toward a conditioned stimulus (CS^+^) and reaching a transparent submerged platform was rewarded with escape from water, whereas swimming toward a non-conditioned stimulus (CS^−^) was not. For every trial, we displayed the CS^+^ in a different arm of the pool according to a Gellerman-like schedule (Treviño et al., 2011; Herrera and Trevino, 2015). Choices were considered as made, once the animals crossed an imaginary line that outlined a decision area with visual access to both CS^+^ and CS^−^ images (Treviño et al., 2012). The animals were allowed to escape from water 30 s after reaching the submerged platform. We repeated the error trials until the animal made a correct choice (max. five error repetitions). These sets of swims, ranging from 1 to 6, constituted a “training unit,” and they involved the same pair of CS^+^/${\text{CS}}_{\text{i}}^{-}$ images (Figure 1B). The water temperature (21 ± 1°C) and room illumination were kept constant throughout the experiments, and the pool was wiped down daily with ~70% ethanol. A detailed description of other crucial elements of the task have been published previously (Treviño et al., 2013; Treviño, 2014).

To create the training stimuli, we downloaded >1000 pictures from the internet and digitally transformed and scaled them to produce black-and-white, equiluminant (~85 lux) images with different irregularities in shape (Treviño et al., 2013). The resulting images where white shapes on a black background, or *vice versa* (*i.e.*, the shape was the only relevant “feature”). We further standardized these images by using a symmetric Gaussian low-pass filter (60 pixel size, 30 pixel standard deviation; ~0.30 cycles per degree [c/d]) to remove all the structural components that surpassed a mouse's visual acuity of ~0.48 c/d (Treviño et al., 2012). We illustrate examples of the resulting stimuli in Figure 1B. We next used the structural similarity index (Wang et al., 2004; Treviño et al., 2013; SSIM) to compare the similarity across all combinations of image pairs (Figure 1B), and selected an eccentric CS^+^ that had one of the highest standard deviations of SSIM values against the rest of the CS^−^ images (Treviño et al., 2013).

We next sorted selected CS^−^ with increasing (blue dots) and decreasing (red dots) SSIM values relative to the CS^+^ (Figure 1C); this constituted the stimulus timeline for visual discrimination training. We also created training programs with increasing and decreasing inter-training unit gradients of CS^+^/${\text{CS}}_{\text{i}}^{-}$ similarity (ΔSSIM). With these oscillating SSIM gradients, we investigated how variations in discrimination difficulty, imposed at the beginning or at the end of training influenced learning. Altogether, we formed nine groups of mice and trained them to discriminate images with either varying (Groups 1–6) or fixed (Groups 7–9) SSIM (Table 1).

**Table 1:** **Groups of mice trained with varying or fixed stimulus similarity conditions**.

**ID**	**Name**	**Mice**	**Training condition**	**SSIM range**
1	SSIM_inc, wide_	9	Increasing SSIM	SSIM from −0.07 to 1
2	SSIM_dec, wide_	9	Decreasing SSIM	SSIM from −0.07 to 1
3	SSIM_inc, narrow_	10	Increasing SSIM	SSIM from 0.04 to 0.39
4	SSIM_dec, narrow_	10	Decreasing SSIM	SSIM from 0.04 to 0.39
5	ΔSSIM_inc, narrow_	10	Increasing ΔSSIM	SSIM from 0.04 to 0.39
6	ΔSSIM_dec, narrow_	10	Decreasing ΔSSIM	SSIM from 0.04 to 0.39
7	SSIM_0.04_	10	Fixed SSIM	SSIM = 0.04
8	SSIM_0.32_	10	Fixed SSIM	SSIM = 0.32
9	SSIM_1_	10	Fixed SSIM	SSIM = 1

The panels in Figures 1C,D are labeled with encircled numbers according to the “IDs” of the groups used in this table. We calculated the average probability of making correct choices (%correct ± %S.E.M) for each trial, and extracted the learning curves of the different groups by using a moving average filter (span = 30 trials, degree = 1; Figure 1D). The data in Figure 1 were published previously (Treviño et al., 2013) and constitute the empirical reference for the mathematical analyses that we developed throughout this work.

### Model fit and parameter estimation

To predict the visual discrimination learning curves, we adapted a basic linear operator model of associative learning (Bush and Mosteller, 1951; Rescorla and Wagner, 1972). The model included a minimum salience threshold for learning, as follows:

(1) $$\frac{dV\left(t\right)}{dt}=\;\{\begin{array}{cc} \alpha \left(t\right)\beta \left[\lambda \left(\alpha \right)-V\left(t\right)\right] & \text{for }\alpha \left(t\right)\geq \alpha_{min}\\ -k_{1}V\left(t\right) & \text{for }\alpha \left(t\right)<\alpha_{min}\&V\left(t\right)\geq V_{\text{min}}\\ 0 & \text{for }\alpha \left(t\right)<\alpha_{min}\&V\left(t\right)<V_{min} \end{array}$$

where *V*(*t*) is the cumulative amount of learning (*i.e.*, the strength of the [CS^+^/${\text{CS}}_{\text{i}}^{-}$]-unconditioned stimulus association); α(*t*) is the *net* stimulus salience (related to the CS^+^/${\text{CS}}_{\text{i}}^{-}$ SSIM, see below); β corresponds to the US salience (0 < β < 1; associated with the intensity of the US), and λ(α) is the asymptote of learning (*i.e.*, maximum retention level). The core model for the *net* salience during training was represented as the sum of *effective* (ϕ) plus *acquired* (ε) salience components (Esber and Haselgrove, 2011):

(2) $$\begin{array}{l} \alpha \left(t\right)=\phi \left(\text{SSIM}\right)+\varepsilon \left(t\right) \end{array}$$

We defined ϕ and ε as either linear or a non-linear functions of stimulus similarity (SSIM) and the associative strength [*V*(*t*)], respectively. We tested for the following salience formulations:

$$\begin{array}{l} \;Model\;Nr.\;Total\;salience\;Effective\;+\;Acquired\\ \;salience(\phi )\;salience(\varepsilon (t))\\ Model\;1\;(\text{Equation 3}):\;\alpha \left(t\right)=\;c_{1}\\ Model\;2\;(\text{Equation 4}):\;\alpha \left(t\right)=\;c_{1}\left(1-SSIM\right)\\ Model\;3\;(\text{Equation 5}):\;\alpha \left(t\right)=\;c_{1}{\left(1-SSIM\right)}^{n_{1}}\\ Model\;4\;(\text{Equation 6}):\;\alpha \left(t\right)=\;c_{1}{\left(1-SSIM\right)}^{n_{1}}+\;c_{2}V{\left(t\right)}^{n_{2}}\\ Model\;5\;(\text{Equation 7}):\;\alpha \left(t\right)=\;c_{1}{\left(1-SSIM\right)}^{n_{1}}+\;c_{2}{\left(\lambda_{max}-V\left(t\right)\right)}^{n_{2}}\\ Model\;6\;(\text{Equation 8}):\;\alpha \left(t\right)=\;c_{1}{\left(1-SSIM\right)}^{n_{1}}+\;c_{2}{\left(\left|\lambda_{max}-V\left(t\right)\right|\right)}^{n_{2}} \end{array}$$

where *c*_1_, *c*_2_, *n*_1_, and *n*_2_ are constants. Also, we defined λ as a sliding logistic function of the *net* salience, because we assumed that the quality of sensory representation would degrade gradually as salience reached α_min_, compromising discrimination and learning (Treviño et al., 2011):

(9) $$\begin{array}{l} \lambda \left(\alpha \right)=\frac{\lambda_{max}}{1+e^{-s\left(\alpha \left(t\right)-\alpha_{min}\right)}} \end{array}$$

where *s* corresponds to the steepness of the sigmoid and α(*t*) is the net salience of the stimulus. With this core model, we assumed that maximum discrimination performance drops toward zero when α ≤ α_min_, but tends toward λ_max_ for high α values and when k_2_ → ∞ (*i.e.*, λ(α) → λ_max_) (Rescorla and Wagner, 1972). Also, we assumed that the *effective* salience of a given stimulus equals that of any other stimulus multiplied by their similarity [ϕ_*i*_(*t*) = ϕ_*j*_(*t*)^*^SSIM_i, j_] (Mackintosh, 1975; Shepard, 1987; Pearce, 1994; Treviño et al., 2011).

**Figure 2:**
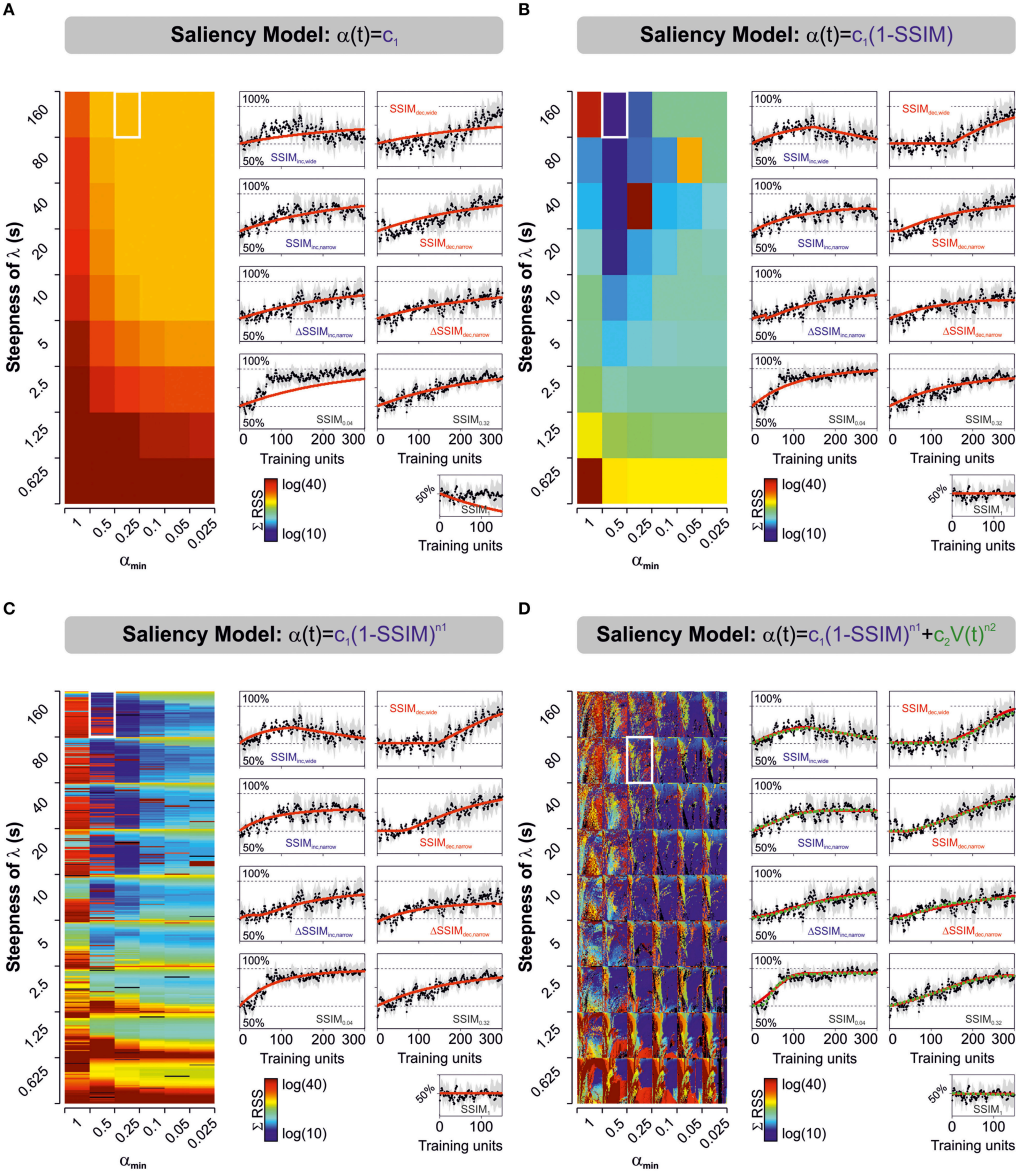
**Predicted learning curves using different parameterizations for stimulus salience**. Predicted learning curves (red lines) fitted to the empirical average choice records from mice trained with varying and constant stimulus similarity. Salience was represented as either a simple constant **(A)**, as a linear **(B)**, or non-linear **(C)** function of stimulus similarity (SSIM), or as the sum of two non-linear functions, one dependent on stimulus similarity, and the other on *V*(t) **(D)**. Color-maps display the sum of squares (ΣRSS) for all nine experimental groups (same color scale for all cases). In **(C)** we mapped for different values of *n*_1_ (*n*_1_ ≥ 0:0.1:6, y-axis), whereas in **(D)** we mapped for *n*_1_ (y-axis) and *n*_2_ (*n*_2_ = 0:0.1:6; x-axis). Common to all groups: β, *c*_1_, *k*_2_, and α_min_. In **(D)**, the red and green lines correspond to the best “unbound” (0 > ϕ ≥ ∞; 0 > ε ≥ ∞) and “bound” (0 > ϕ ≥ 1; 0 > ε ≥ 1) solutions, respectively. The best parameter fits for the unbound solution were β = 0.0154; *k*_1_ = 0.0014; *V*_min_ = 0.55; λ_max_ = [1, 0.97, 0.77, 0.95, 0.84, 0.85, 0.70, 0.90, 0.93]; *s* = 80; α_min_ = 0.25; *c*_1_ = 0.3827; *n*_1_ = 1.5; *c*_2_ = [0.31, 4.01, 12.91, 2.15, 2.92, 1.41, 0.00, 3.36, 9.56]; *n*_2_ = 6, whereas those for the bound solution were β = 0.0140; *k*_1_ = 0.0014; *V*_min_ = 0.55; λ_max_ = [1, 0.95, 0.77, 0.95, 0.85, 0.86, 0.70, 0.90, 0.93]; *s* = 80; α_min_ = 0.25; *c*_1_ = 0.6762; *n*_1_ = 2.7; *c*_2_ = [0.27, 4.57, 10.88, 2.30, 2.64, 1.08, 0.00, 3.63, 7.11]; *n*_2_ = 5.6. The arrangement of the panels with the learning curves is identical to the one described for Figures 1C,D.

To solve how the associative learning model would fit the experimental learning curves from the mice, we implemented nonlinear programming routines using standard optimization tools from MATLAB (The MathWorks, Inc., USA). With this approach, we aimed finding the best model parameters that produced learning curves that mimicked the empirical correct choice records from the trained mice. Thus, we employed the 2nd order Runge-Kutta method to solve Equation (1) at discrete intervals. Next, to find the model parameters that minimized the residual sum of squares (RSS) between experimental data and the model fit, we applied a generic nonlinear multivariable optimization algorithm (sequential quadratic programming, SQP). We used a step-size = 1 and initialized *c*_1_ and *c*_2_ with a zero value for all groups. Importantly, β, *c*_1_, *n*_1_, *k*_2_, and ϕ_min_ adopted identical optimized values for all groups. We illustrate some of the predicted learning curves in Figure 2.

To compare the “goodness of fit” among the different models, we adopted the Akaike Information Criterion [*AIC* (Burnham and Anderson, 2002)]. The *AIC* is a metric that seeks a model with a high-quality fit to the observed values, yet with as few free parameters as possible. The second order *AIC* (*AICc*) compensates for sample size by increasing the relative penalty for fits with small data sets:

(10) $$\begin{array}{l} {AIC}_{C}=n\cdot ln\left(\frac{RSS}{n}\right)+2K+\frac{2K\left(K+1\right)}{\left(n-K-1\right)} \end{array}$$

where *RSS* is the residual sum of squares between the model and the empirical data, *n* is the number of observations (*i.e.*, sample size), and *K* is the number of free parameters. We ranked all models by taking the best approximation with the lowest (most negative) *AIC*_*C*_ value. Next, we calculated the Δ*AICc* as the difference between the best model (smallest *AICc*) and the *AIC*_*C*_ for each model (*i.e.*, the best model has a Δ*AIC*_*C*_ of zero). Finally, to calculate the Akaike weights (*w*_*i*_), we took the relative likelihood of each prediction and divided it by the sum of these values across all models, as follows:

(11) $$w_{i}=\frac{e^{-\text{0}.\text{5}\cdot △AIC_{C}}}{\sum_{r=1}^{R}e^{-\text{0}.\text{5}\cdot △AIC_{C,r}}}$$

These weights had a normalized value falling between 0 and 1, corresponding to the probability that a given model becomes the best approximation and with the sum of all weights being 1. Overall, these coefficients take into account how well the model fits the data (using the *RSS*), favoring descriptions with fewer free parameters, as it penalizes the number of fitted parameters (*K*).

We displayed results as averages ± S.E.M and employed parametric and non-parametric statistical tests with a significance set at *p* < 0.05.

## Results

### Learning curves for mice trained with varying similarity

We trained mice to learn to discriminate combinations of CS^+^/CS^−^ images (Figure 1A). The stimuli offered varying degrees of structural similarity (SSIM; Wang et al., 2004; Treviño et al., 2013; Figure 1B). The ${\text{CS}}_{\text{i}}^{-}$ stimuli were either exchanged on every trial or remained fixed during acquisition (Figure 1C). The first two experimental groups were trained with stimuli sorted with increasing (SSIM_inc, wide_; Figure 1C_1_, blue dots) or decreasing (SSIM_dec, wide_; Figure 1C_2_, red dots) similarity values. That is, this training consisted of sustained positive or negative SSIM gradients ranging from −0.07 to 1. The SSIM_inc, wide_ group displayed an initial growth in **its** average correct choice level, peaking at 88.5 ± 9.3% around the middle of training and then decreasing when SSIM > 0.30 (Figure 1D_1_). By contrast, the decreasing similarity group (SSIM_dec, wide_) displayed a slower onset of correct choice behavior and higher maximal performance of 98.7 ± 9.2% (Figure 1D_2_).

We next trained two additional groups of mice (SSIM_inc, narrow_: Figure 1C_3_ and SSIM_dec, narrow_: Figure 1C_4_) in which the maximum similarity was kept below SSIM = 0.39. This training ensured that CS^+^/CS^−^ discriminability remained fully uncompromised during acquisition (Treviño et al., 2013). The correct choice level reached a plateau at maximal performance in the SSIM_inc, narrow_ group (Figure 1D_3_) while the SSIM_dec, narrow_ group displayed a slower onset for correct choice and a higher average peak performance at the end of training (Figure 1D_4_). We also tested training **mice** with increasing (ΔSSIM_inc, narrow_: Figure 1C_5_) or decreasing (ΔSSIM_dec, narrow_: Figure 1C_6_) oscillating gradients in similarity. Both ΔSSIM groups displayed similar learning rates and maximum performance (Figure 1D_5__,__6_).

Finally, we trained mice with a fixed similarity of SSIM_0.04_ = 0.04 (Figure 1C_7_), SSIM_0.32_ = 0.32 (Figure 1C_8_), and SSIM_1_ = 1 (Figure 1C_9_). These groups allowed us to investigate how different, but fixed similarities, led to specific learning rates and peak discrimination performances (Rescorla and Wagner, 1972). As expected, the SSIM_1_ mice failed to learn to discriminate (Figure 1D_9_), but the other two groups showed increasing learning rates when trained with SSIM_0.32_ (Figure 1D_8_) and SSIM_0.04_ (Figure 1D_7_), respectively. Therefore, the learning rate increased by lowering stimulus similarity whereas shape and maximum retention level of the learning curves were determined by the sign and slope of the SSIM gradients during training (Treviño et al., 2013). Also, the changes in choice performance through learning were slow and retained across daily sessions (Karni and Sagi, 1993). The learning curves illustrated in Figure 1D were described in detail previously (Treviño et al., 2013) and serve as the empirical reference or “ground truth” for the mathematical analyses performed in the following sections.

### Testing multiple representations for stimulus salience

We aimed to explore how the changes in stimulus similarity during acquisition could explain the empirically observed learning curves (Figure 1D). We used a simple linear operator model of associative learning aiming to predict the evolution of the associative strength (*V*(*t*); Bush and Mosteller, 1951; Rescorla and Wagner, 1972; Treviño et al., 2011). Our adaptation of this core predictive model includes important considerations. It assumes that the discrimination process does not lead to learning when stimulus salience is below a minimum *effective* salience threshold [α(*t*) ≤ α_min_, *V*(*t*) < *V*_*min*_]. Yet, it will produce learning when α(*t*) > α_min_. Also, we defined the asymptote of learning (λ) as a sliding logistic function of α (see Materials and Methods), as proposed previously (Treviño et al., 2011). We did so because we reasoned that the quality of sensory representation should degrade gradually as the salience reaches α_min_, compromising discrimination and learning (Treviño et al., 2011, 2013). The resulting equations predict that, at any given trial, the change in *V*(*t*) will be proportional to the product of the CS^+^/CS^−^ and the US saliencies acting linearly on the difference between the asymptote of learning [λ(α)] and *V*(*t*) (see Materials and Methods). In the simplest case, if α were a constant, *V*(*t*) would increase in a negatively accelerated manner, as *V*(*t*) approached λ(α) (Rescorla and Wagner, 1972).

One remarkable feature of our experimental data set is the fact that we changed the stimulus similarity during training (Groups 1–6; Figure 1C). In the next sections, we will illustrate how we explored the predictive power of different mathematical representations for stimulus salience. More concretely: our main hypothesis was that the *net* stimulus salience could be represented as the sum of *effective* (ϕ) plus *acquired* (ε) components (Esber and Haselgrove, 2011). In the equations we used, the *effective* salience (ϕ) derived explicitly from the visual properties of the stimulus (*i.e.*, a function of SSIM) whereas the *acquired* salience [ε(*t*)] depended on the reinforcement history (Mackintosh, 1975; Pearce and Hall, 1980).

We tested and compared the predictive power of six models that represented stimulus salience with different equations (see Materials and Methods). For the first three models, we defined the net salience as an *effective* salience component only [*i.e.*, α(*t*) = ϕ(SSIM)]. We set such *effective* salience as being either a simple constant (Model 1; Rescorla and Wagner, 1972), or as a constant that depended linearly (Model 2), or non-linearly (Model 3) on stimulus similarity (SSIM; Mackintosh, 1975; Pearce and Hall, 1980; Le Pelley, 2004). This description for *effective* salience (ϕ) constitutes an objective approach to quantify the structural differences among visual stimuli (Wang et al., 2004; Treviño et al., 2013), and it is consistent with the fact that it should be positively correlated with α (Mackintosh, 1975). For the last three salience formulations, we added a second component representing the *acquired* salience (ε). Model 4 involved a linear (*n*_2_ = 1) or a non-linear (*n*_2_ ≠ 1) function of the amount of learning [*V*(*t*)], allowing ε to grow monotonically with learning. Formally, this model implied that the ε would grow as a stimulus became a better predictor of an outcome (*i.e.*, leading to a smaller prediction-error; Mackintosh, 1975). In contrast, for models 5 and 6, we defined ε in such a way that it would decrease with learning [because *V*(*t*) tends toward λ_max_ with training repetitions]. These two last models implied that the *acquired* salience would decrease with learning as the outcome of a trained stimulus became predictable (Pearce and Hall, 1980).

We employed non-linear programming techniques to fit all the models to the experimental data (see Materials and Methods). This approximation aimed to find those model parameters that minimized the residual sum of squares (*RSS*) between the experimental data and the model fits. We quantified the “goodness of fit” by applying the solver to the empirical learning curves from the nine experimental groups and then calculating the sum of *RSS* (Σ*RSS*) using different saliency descriptions (Figure 2). The color maps in Figure 2 illustrate the Σ*RSS* values mapped for different λ(α) slopes and α_min_ values. In some cases, we tested for various *n*_1_ values (Model 3; *n*_1_ = 0:0.1:6; Figure 2C) or the combination of multiple *n*_1_ (y-axis) and *n*_2_ values (x-axis; Model 4; *n*_1_ = 0:0.1:6, *n*_2_ = 0:0.1:6; Figure 2D). To enable valid comparisons, we applied the same color scale for all panels. The predicted learning curves (in red) correspond to the solutions that produced lowest Σ*RSS* for each model using “unbound” salience ranges (0 > ϕ ≥ ∞; 0 > ε ≥ ∞). The green dotted line in Figure 2D corresponds to the solution to Model 4 with ‘bound’ salience conditions (*i.e.*, 0 > ϕ ≥ 1; 0 > ε ≥ 1). We provide the optimized parameters for the two best solutions in the legend of Figure 2. The predicted learning curves for salience models 5 and 6 are not illustrated.

### A model with effective plus acquired salience predicts the learning curves

**Figure 3:**
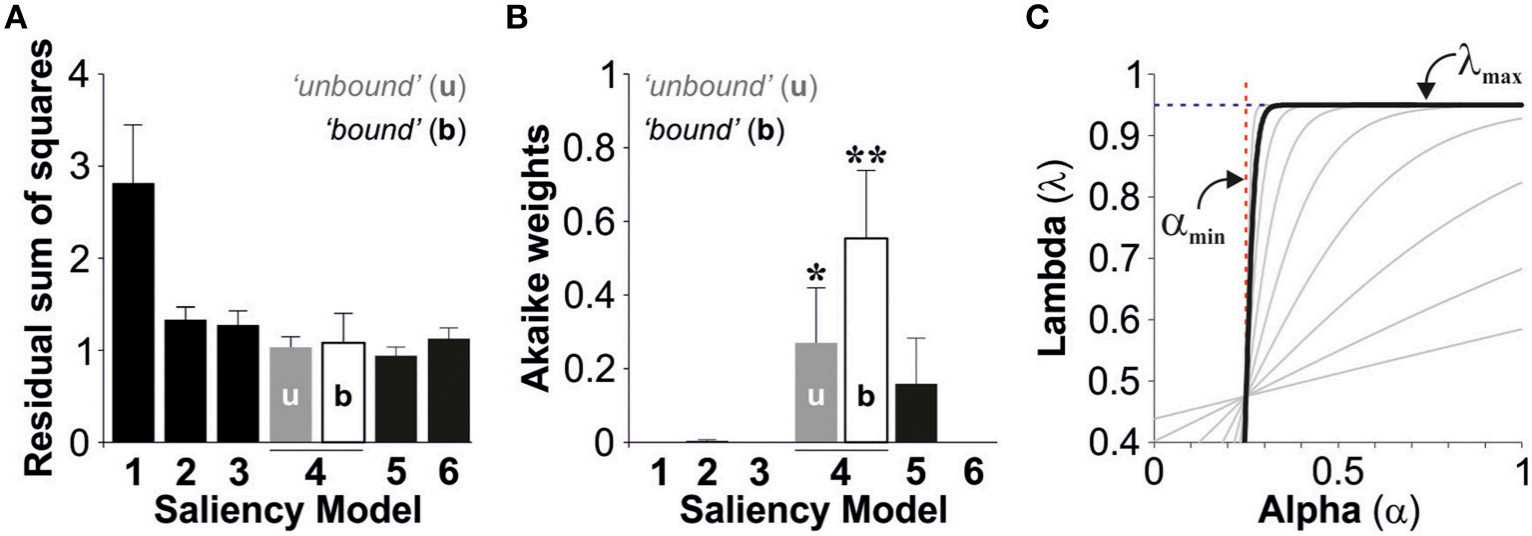
**Best model selection**. The bar plots display the group average residual sum of squares **(A)** and the Akaike weights **(B)** for the saliency models tested (Average ± S.E.M). **(C)** Plot of lambda *vs.* alpha using the best parameter fits described in Figure 2 (“bound” conditions). The pattern resembles a Heaviside step function suggesting that lambda does not depend on alpha.

We used the second order Akaike Information Criterion (*AICc*) to compare and select the best description, from the models tested, for stimulus salience. Using an information theory approach (Burnham and Anderson, 2002), we seeked a model that presented a good fit to the observed data (Figure 3A), yet with as few free parameters as possible. This method also takes into account sample size by increasing the penalty for small data sets (see Materials and Methods). Finally, we calculated the Akaike weights (*w*_*i*_) for each model (Table 2 and Figure 3B). Individual weights had a value between 0 and 1, corresponding to the probability that a given model constitutes the best approximation (Σw = 1), which thus provides a quantitative estimation of the relevance of the models under consideration. The saliency models 1, 2, 3, and 6 had minor Akaike weights (<1%). However, the fourth salience model captured the strongest weights with 27 ± 15% with unbound conditions and 56 ± 19% with bound conditions. The fifth model captured 16 ± 12% of the total weight (paired *t-tests* against models 1,2,3, and 6, ^*^p < 0.05, ^**^p < 0.01; Figure 3B). Therefore, the Model 4 (“bound” conditions) provided the best approximation from all models tested (One-way ANOVA multi-comparison test; *F*_(6, 55)_ = 4.80, *p* < 0.001).

**Table 2:** **Comparison of model fits with different free parameters**.

**Group averaged values**					
**Model**	**K**	**RSS**	**AIC**_C_	Δ_i_	**w**_i_
1	4	2.82 ± 0.64	−1353.40 ± 120.76	475.91 ± 109.06	0.00 ± 0.00
2	5	1.33 ± 0.16	−1516.20 ± 83.19	313.11 ± 111.33	0.01 ± 0.01
3	6	1.28 ± 0.15	−1523.42 ± 84.11	305.88 ± 108.78	0.00 ± 0.00
4	7	1.04 ± 0.12	−1583.89 ± 92.83	245.42 ± 101.92	0.27 ± 0.15
5	7	1.09 ± 0.33	−1734.10 ± 191.00	95.21 ± 47.82	0.56 ± 0.19
6	7	0.95 ± 0.10	−1604.58 ± 95.61	224.72 ± 96.45	0.16 ± 0.12
7	7	1.13 ± 0.14	−1560.93 ± 91.53	268.38 ± 101.30	0.00 ± 0.00

We arranged in columns (from left to right): Model number, number of free parameters, the ΣRSS (sum of residual sum of squares), the second order Akaike coefficients, the difference between model with lowest AIC_C_ (Δ_i_) and Akaike weights (w_i_).

We next solved lambda (Equation 9) by using the best parameter fits with the best model (model 4, bound conditions). The obtained relationship between lambda and alpha resembled a Heaviside step function indicating that these two variables are independent from each other (Figure 3C). Therefore, this means that we can treat lambda as a constant, yet this does not impair our conclusions because the equations we used to describe lambda covered this scenario (λ(α) → λ_max_ when s → ∞).

### Evolution of *acquired* salience with learning

**Figure 4:**
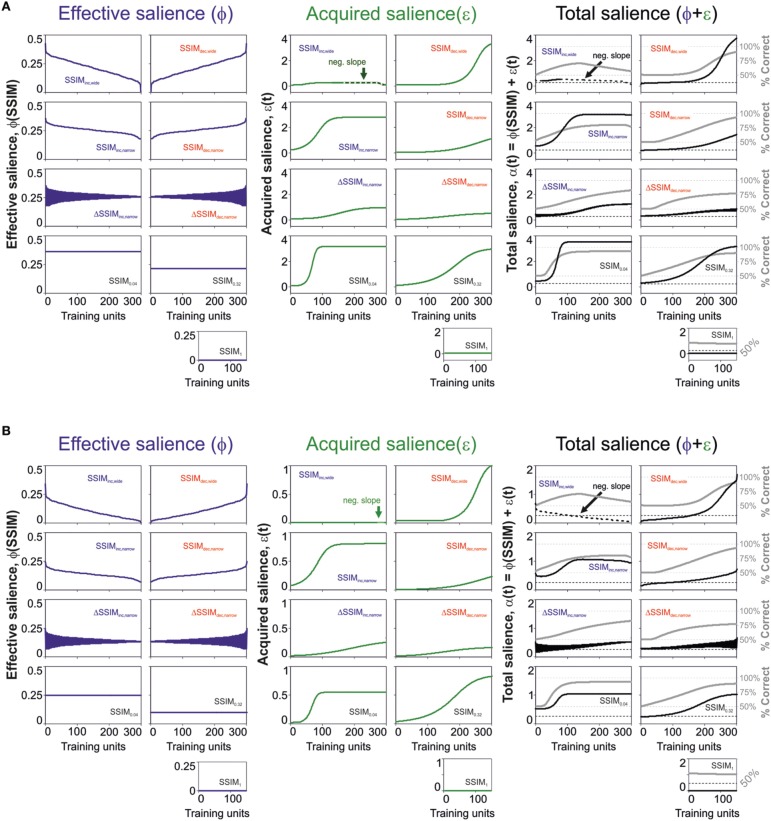
**Changes in effective and acquired salience through training**. Dynamic changes in *effective* (blue lines), *acquired* (green lines), and total (black lines) salience for the best predictive model parameters using “unbound” (0 > ϕ ≥ ∞; 0 > ε ≥ ∞; **A**) and “bound” (0 > ϕ ≥ 1; 0 > ε ≥ 1; **B**) salience conditions. The gray lines on the right panels correspond to the predicted associative strength curves.

The previous results revealed that a salience representation using *effective* plus *acquired* salience provided a better description of the learning curves compared to models considering the *effective* salience only. Yet, how does the *acquired* salience evolve with acquisition? Salience could increase or decrease depending on how good or bad a stimulus predicts its consequences (Mackintosh, 1975). Alternatively, the salience could decrease (or increase) as the stimulus becomes a better (or worse) predictor of its consequences (Pearce and Hall, 1980). To distinguish these two possible scenarios in our data, we solved the salience equations employing the best parameter fits with the best predictive model. In Figure 4, we illustrate the numeric solutions for the *effective* [ϕ(SSIM); left], *acquired* [ε(*t*); middle] and total salience [α(*t*) = ϕ(SSIM) +ε(*t*); right] with “unbound” (upper row; Figure 4A) and “bound” (lower row; Figure 4B) conditions. The trajectories for these saliency variables were quite similar for both solutions. As expected, the *effective* salience ϕ varied with an opposite slope with respect to changes in similarity (SSIM), because it is proportional to 1-SSIM (see Materials and Methods). The *acquired* salience, on the other hand, increased monotonically for all training programs except SSIM_inc, wide_ (Mackintosh, 1975). For this group, the *acquired* salience had a region of negative slope linked to the systematic drop in *effective* salience and the decay of *V*(*t*) (green arrow, Figures 4A,B, middle). As a result, almost for all groups, the total salience grew slowly (on average) because it consisted of the sum of *effective* plus *acquired* salience. Only the SSIM_inc, wide_ group had a minor portion with monotonic reduction in *net* salience, because it combined the decay for both the *effective* and the *acquired* salience. These results are consistent with the idea that the *acquired* salience grows as a stimulus becomes a better predictor of an outcome (Mackintosh, 1975).

### The learning trajectory determines the maximum acquired salience

**Figure 5:**
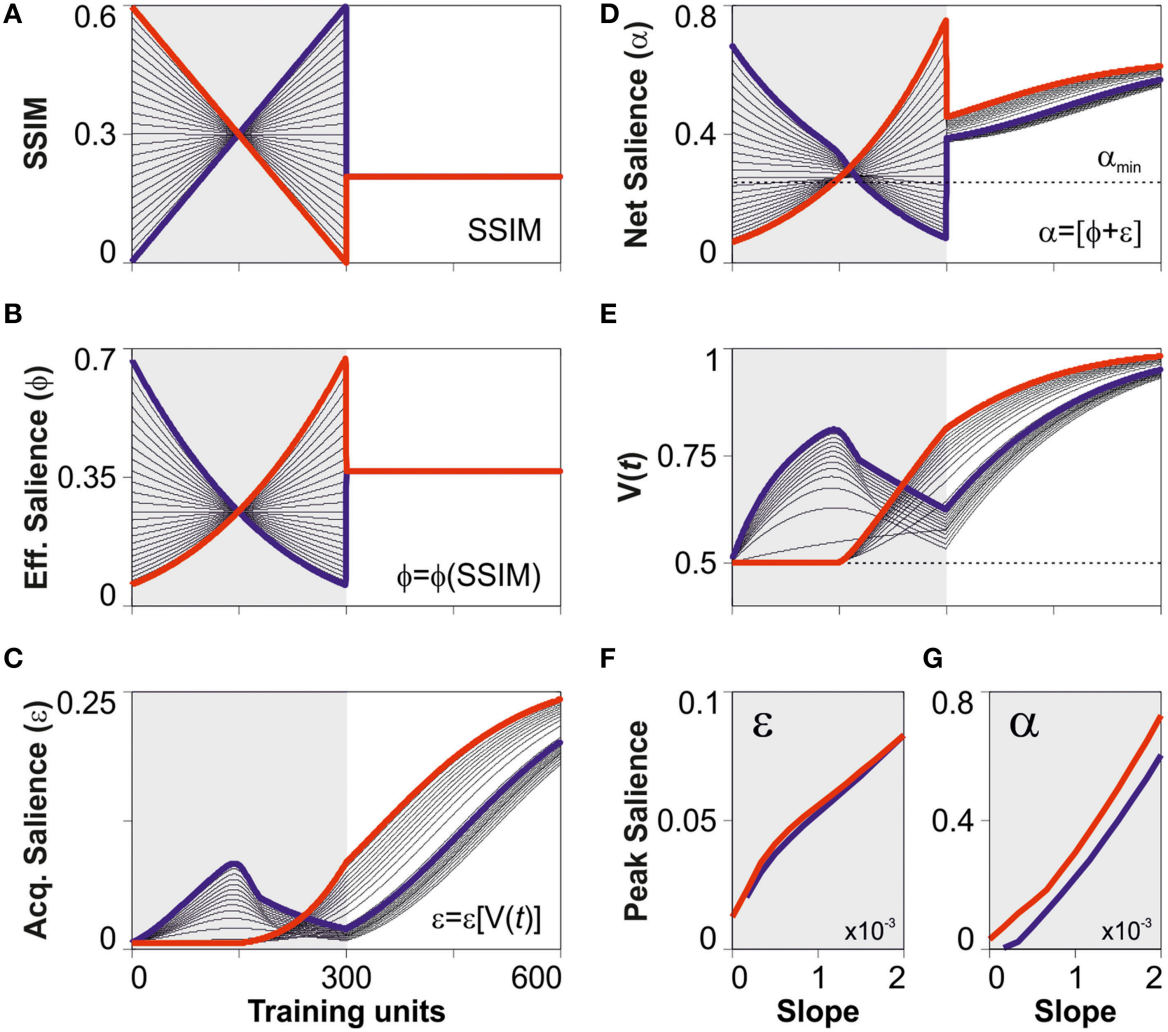
**Differences in peak salience for training programs with identical stimuli**. **(A)** Synthetic creation of SSIM training programs consisting of a variable SSIM epoch followed by a constant one. The first, “variable” epoch (from trial 1 to 300) was created using linear ramps with positive (blue) and negative (red) SSIM slopes. **(B)** These arrangements consist of the same stimuli, sorted in ascending (blue) or descending (red) order, respectively. The second, “constant” epoch (trials 301–600) consisted of training with the same constant SSIM, one that led to a net salience α(SSIM) > α_min_. Solving Equation (7) (see Materials and Methods) we display the *effective* **(B)**, *acquired* **(C)**, and *net* **(D)** salience. To simulate discriminative training, we took the optimized parameters from the best predictive model displayed in Figure 2D (“bound” salience; green dotted line) and numerically solved Equation (1) (see Materials and Methods). We illustrate the predicted learning curves in panel **(E)**. The overall differences in the learning curves can be explained by the differences in *net* **(G)** but not in *acquired* **(F)** peak salience during training.

In this last section, we explored some of the possible predictions derived from the dual salience model. To do so, we defined a virtual training program based on two epochs. For the first epoch (trials 1–300), we used linear arrays of SSIM changing through discrete training steps (step-size = 1). These numbers represented training stimuli with different similarities yet the programs consisted of the same stimuli, sorted in ascending (blue), or descending (red) order, respectively. Note that subtracting these two scenarios maximizes the relative difference in SSIM between training programs. The second epoch (trials 301–600), consisted of training with constant SSIM ensuring a total salience above α_min_. To simulate the discriminative learning, we took the virtual SSIM programs described above (Figure 5A) and, using the optimized parameters from the best predictive model (*i.e.*, model 4 “bound” conditions), we solved Equation (1) by using the Runge-Kutta method with discretized time-steps (see Materials and Methods). We then extracted the values for the *effective* (Figure 5B), *acquired* (Figure 5C), and *net* (Figure 5D) saliencies, and also for the associative strength (Figure 5E). As expected, the temporal arrangement of SSIM determined the shape of the learning curves. When stimuli had a total salience below α_min_, they were undetectable, *V*(*t*) did not increase, and the learning curves decayed toward chance level due to the lack of reinforcement [0 ≤ *V*(*t*) ≤ 1]. Notably, when similarity was held constant during the second epoch, the group trained with positive SSIM changes always performed below the group trained with negative SSIM gradients (Figure 5E). To further explore these differences, we took the peak values for the *acquired* and *net* saliencies observed during the first 300 trials (shaded region in the panels) and plotted them against the SSIM slope during training. Interestingly, although the maximum *acquired* salience was similar for the groups trained with positive and negative SSIM slopes (Figure 5F), the peak *net* salience was always bigger for negative (red lines) than for positive (blue lines) SSIM gradients (Figure 5G). This demonstrates that the differences in the trajectories in associative strength were due to the different arrangements of *effective* salience during training leading to a bigger *net* saliencies for the group trained with negative SSIM. Such differences in *net* salience provide a satisfactory explanation as to why the temporal arrangement of stimuli with the same *effective* salience can produce profoundly different learning curves (Treviño et al., 2013).

## Discussion

We adapted a mathematical model in order to predict the choice records from nine groups of mice trained with heterogeneous visual stimuli (Treviño et al., 2013). Specifically, we compared the predictive power of a simple associative learning rule coupled to six different saliency descriptions with the idea of gaining insight into the salience mechanisms involved in the learning process. Several studies have assumed that the contribution of the conditioned stimuli to learning is fixed (Bush and Mosteller, 1951; Rescorla and Wagner, 1972). Here, we explored a complementary view: we tested whether our empirical data could be explained by learning rules that involved a more flexible representation for stimulus associability, as suggested by other authors (Mackintosh, 1975; Pearce and Bouton, 2001; Le Pelley, 2004; Esber and Haselgrove, 2011). Indeed, we coupled our learning model to functions that allowed us to represent the stimulus salience in different relevant ways. By using the Akaike Information Criterion (*AIC*), we found that the best predictive model was one in which we defined the *total* salience as the sum of *effective* plus *acquired* components. The *effective* salience was a fixed quantity that depended only on the physical attributes of the stimuli whereas the *acquired* salience depended on how the associative strength changed during acquisition. This second component allowed the *net* salience to change as a result of learning about the outcome of the trained stimuli (Lawrence, 1949, 1950; Mackintosh, 1975; Esber and Haselgrove, 2011).

The exact definitions for *effective* and *acquired* saliencies were relatively unimportant to us because there are multiple other ways in which they could be formulated. Yet, we avoided some specific combinations of variables due to analytical reasons. For example, variables that are introduced into formulas as multiplicative factors are not structurally identifiable. This is because there is an infinite number of possible values that such variables could adopt to solve the mathematical problem: “a change in one parameter can be compensated by a proportional shift in the other one, still producing a satisfying fit between experimental data and model predictions” (Dochain and Vanrolleghem, 2001).

The structural similarity index (SSIM; Wang et al., 2004) constitutes a useful quantitative tool for assessing perceptual similarity both in humans (Wang et al., 2004) and mice (Treviño et al., 2013). We used this index to compare the similarity among images and defined the *effective* salience as a non-linear function of it. Moreover, we assumed that the *effective* salience of a stimulus could be represented by the salience of any other stimulus times the similarity between them (Mackintosh, 1975; Pearce, 1994; Treviño et al., 2011). This notion implied that the changes in associative strength were extracted through generalizations across stimuli (Lawrence, 1952; Shepard, 1987; Dosher and Lu, 2007; Cleland et al., 2009). Behavioral generalization is a process in which similar stimuli become associated with the same contingency and has been used to assess perceptual similarity in diverse animal models (Lawrence, 1949, 1952; Pearce, 1987; Shepard, 1987; Dosher and Lu, 2007). The psychometric function that describes how different stimuli group together is called generalization gradient and decreases with perceptual similarity distance (Shepard, 1987). It would be of particular interest to explore how the changes in *effective* salience during training influence the shape of the generalization gradient.

Many learning models propose that conditioned stimuli are processed with constant salience (*v.gr.* Rescorla and Wagner, 1972). In nature, however, salience is variable because context and experience change dynamically. Indeed, there is ample evidence that saliency signals depend on prior experience as they can be acquired *via* contiguity (Lawrence, 1949; Sutherland and Mackintosh, 1971). Recently, Esber and Haselgrove (2011) developed an attentional model based on *effective* (*i.e.*, stimulus-driven) plus *acquired* salience. Inspired by their model, we defined our equations in such a way that the *acquired* salience would evolve with the associative history of trained stimuli (Mackintosh, 1975; Pearce, 2008; Esber and Haselgrove, 2011). This formulation allowed the *net* stimulus salience to grow or decay slowly, depending on how the subjects learned about the predictive power of the stimuli (*i.e.*, the learning trajectory; Mackintosh, 1975). Our analytical results revealed that, as suggested previously, training with a fixed stimulus increased the *acquired* salience through learning (Mackintosh, 1975). One important implication of this explanatory frame is that stimuli with the same *effective* salience (*i.e.*, identical stimuli) can have different *net* saliencies and *vice versa* (*i.e.*, different stimuli having the same *net* salience).

Theories of selective attention propose that learning about a stimulus requires attending to that particular stimulus on the first place. They also claim that a spatial “saliency map” can describe the salience of an entire visual scene, allowing the detection of locations with distinctive visual attributes (Gottlieb et al., 1998; Itti and Koch, 2001). The activity of some neurons in the visual cortex is involved in the computation of elementary features of the stimulus including a “preliminary” saliency map (Itti and Koch, 2001; Li, 2002). Such an initial salience computation in the visual cortex is a “bottom-up”, stimulus-driven signal that contains information about how different is a stimulus from its surroundings (Koch and Ullman, 1985; Ahissar and Hochstein, 1993; Desimone and Duncan, 1995; Reynolds et al., 2000; Li, 2002; Baker et al., 2004; Gutnisky, 2009). Our results support this view that, indeed, the physical properties of a stimulus determine its initial salience, but then, along longer time scales, the stimulus salience will also evolve with experience. We propose that the *effective* stimulus salience is thus a perceptual consequence of a complex interaction of the target stimulus with other surrounding stimuli. This information is then processed by cortical sensory neurons, which change their response properties based on prior experience (Gilbert et al., 2009; Sasaki et al., 2010). Other brain areas involved in processing salience signals include the ventral visual pathway (Mazer and Gallant, 2003; Serences and Yantis, 2007), the frontal eye fields (FEF; Thompson and Bichot, 2005; Serences and Yantis, 2007), the orbitofrontal cortex (Lucantonio et al., 2012; Ogawa et al., 2013) and some subcortical structures such as the superior colliculus (SC; Kustov and Robinson, 1996) and the pulvinar (Robinson and Petersen, 1992).

A multitude of factors can trigger changes in stimulus salience. Rescorla and Wagner (1972) acknowledged the fact that the salience of a stimulus could decrease through pre-exposure to the stimulus. This pre-exposure effect to the CS^+^ is generally referred to as “latent inhibition” (LI) and has been demonstrated in a number of animal species (Lubow and Moore, 1959; Le Pelley, 2004). One common interpretation for LI is that it arises from a reduced stimulus salience as a result of an experience with the stimulus without consequence in the non-reinforced “pre-exposure” phase (Lubow and Moore, 1959). Such a reduction in the learning rate produced by LI could reflect the capacity of individuals not to attend to, or to ignore, stimuli that predict no significant consequences (Weiner, 2003). Interestingly, LI is disrupted in rodents injected with amphetamines (which promote dopamine [DA] release) leading to psychotic symptoms, and this is reversed by treatment with antipsychotic drugs (which potentiate LI). Pharmacological disruption LI is thus considered to provide an animal model of some of the symptoms of schizophrenia (Weiner, 2003).

To make optimal decisions, animals must integrate information about their previous actions and compare them with their current needs (Lucantonio et al., 2012). They are particularly receptive to events that violate their expectations, which in turn facilitate associative learning (Mackintosh, 1975; Pearce and Bouton, 2001). Such a mismatch between expectancy and experience constitutes a prediction-error, driving learning through the allocation of attention to specific stimuli in the environment (Rescorla and Wagner, 1972; Mackintosh, 1975). Prediction errors and expectancies of reward are represented by midbrain DA neurons (Schultz, 2013) which send projections to the prefrontal and orbitofrontal cortices (Gottfried et al., 2003; Corlett et al., 2007; Mainen and Kepecs, 2009). A current proposal is that a dysregulated, hyperdopaminergic state in patients with psychiatric disorders like schizophrenia (and also in drug addiction), leads to disrupted prediction-error processing and aberrant assignment of salience. From this point of view, psychotic states are preceded by an exaggerated release of DA providing motivational significance to irrelevant stimuli (Kapur, 2003; Corlett et al., 2007; Lucantonio et al., 2012). Notably, psychostimulant agents that trigger DA release (*v.gr.* amphetamines) are associated with *de novo* psychosis, whereas antipsychotics that reduce DA transmission, assist in the resolution of the symptoms (Kapur, 2003; Weiner, 2003). For these reasons, we believe that understanding how salience is acquired will be a fundamental step to understand sensory information processing in normal and pathological conditions. The analytical tools that we developed in this work could enable the characterization of aberrant salience acquisition in animal models of schizophrenia.

## Author contributions

MT conceived, designed and performed the data analysis. MT made figures and wrote the manuscript.

## Funding

Support was provided from the Mexican Consejo Nacional de Ciencia y Tecnología (CONACYT: 220862, 07384, 251406).

### Conflict of interest statement

The author declares that the research was conducted in the absence of any commercial or financial relationships that could be construed as a potential conflict of interest.
